# Does Robotic Spine Surgery Add Value to Surgical Practice over Navigation-Based Systems? A Study on Operating Room Efficiency

**DOI:** 10.3390/medicina60122112

**Published:** 2024-12-23

**Authors:** Pirateb Paramasivam Meenakshi Sundaram, Daniel Yang Yao Peh, Jane Wenjin Poh, Guna Pratheep Kalanchiam, Wayne Ming Quan Yap, Arun-Kumar Kaliya-Perumal, Jacob Yoong-Leong Oh

**Affiliations:** 1 Division of Spine, Department of Orthopaedic Surgery, Tan Tock Seng Hospital, Singapore 308433, Singapore; pirateb.sundaram@mohh.com.sg (P.P.M.S.); guna.hytech@gmail.com (G.P.K.); wayne_mq_yap@ttsh.com.sg (W.M.Q.Y.); 2 Lee Kong Chian School of Medicine, Nanyang Technological University, Singapore 636921, Singapore; dpeh004@e.ntu.edu.sg (D.Y.Y.P.); jane0019@e.ntu.edu.sg (J.W.P.); 3 Department of Orthopaedics, Meenakshi Mission Hospital and Research Centre, Madurai 625107, India; 4 Rehabilitation Research Institute of Singapore, Lee Kong Chian School of Medicine, Nanyang Technological University, Singapore 308232, Singapore

**Keywords:** computer-assisted surgery, pedicle screw, robotic surgical procedures, spinal fusion, spondylosis

## Abstract

*Background and Objectives:* Spine surgery has undergone significant advancements, particularly with regard to robotic systems that enhance surgical techniques and improve patient outcomes. As these technologies become increasingly integrated into surgical practice, it is essential to evaluate their added value and cost savings. Hence, this study compared robot-assisted and navigation-based spine surgery, focusing on surgical efficiency. *Materials and Methods:* We conducted a single-center, retrospective cohort study of patients undergoing single- and double-level transforaminal lumbar interbody fusion (TLIF) and oblique lumbar interbody fusion (OLIF) surgeries. Patients were divided into two groups: those who had robot-assisted and navigation-based surgeries, stratified by surgery type (TLIF or OLIF) and fusion levels (one or two). A comparative analysis of factors related to surgical efficiency, including operative duration, blood loss, and length of hospital stay, was conducted. *Results:* Our results showed a statistically significant reduction in operative duration for robot-assisted one- and two-level OLIF cases, with average time savings of 50 and 62 min, respectively, compared to navigation-based surgery. These time savings translated to an estimated cost reduction of SGD 1500 for the hospital for each patient for a two-level OLIF procedure and could be higher as the number of operated levels increase. *Conclusions:* These results indicated that robot-assisted spine surgery offers superior surgical efficiency and cost savings, particularly with increased numbers of surgical levels. As robotic technologies evolve, their integration into spine surgery is justified, promising improved patient outcomes and cost-effectiveness.

## 1. Introduction

Spine surgery has undergone significant advancements over the past few decades, with innovations in technology playing a pivotal role in transforming the surgical techniques used in hopes of better patient outcomes [1,2]. Robotic systems are the latest innovation in the field of spine surgery involving real-time guidance that allows for the precise placement of screws along a trajectory determined by preoperative imaging and planning [3]. Originally introduced in the early 2000s with limited features, robotic systems have undergone significant updates that have enhanced their capabilities, making them integral to modern spine surgery practices and highly relied upon by spine surgeons today [3]. The robot essentially acts as an enhancement to real-time computer navigation-based screw placement technology. Its added value lies in the ability to place the screw without constantly adjusting the trajectory while monitoring the navigation screen, which is necessary during navigation-based surgery. Instead, the screw can be placed directly through the robotic arm, streamlining the process. This approach is pedagogic in nature, as it simplifies the procedure and allows for more intuitive, hands-on learning, making it easier for early career surgeons to perform and master the technique.

Beginning with SpineAssist (Mazor Robotics Ltd., Caesarea, Israel), the first robotic system for spinal surgery, we have now reached the third-generation Mazor X Stealth Edition (Medtronic Inc., Dublin, Ireland), which integrates the Mazor X system with Medtronic’s Stealth intraoperative navigation technology [4]. Other systems include the ROSA Surgical Robot (Zimmer-Biomet, Warsaw, IN, USA), the ExcelsiusGPS Robotic Guidance and Navigation System (Globus Medical Inc., Audubon, PA, USA), the CUVIS Spine (Curexo Inc., Seoul, Korea), the CirQ Robotic Arm (BrainLAB AG, Munich, Germany), and the TIVAVI system, co-designed by Beijing Jishuitan Hospital and Beijing Tinavi Medical Technologies Co., Ltd. (Beijing, China) [5].

The advantages robotic systems bring to spine surgery are numerous. In addition to assisting surgeons in achieving the precise placement of pedicle screws and significantly reducing the risk of implant malposition [6], these robotic systems also provide greater control in navigating complex spinal anatomy, potentially leading to shorter operative times and a reduced risk of complications [6,7,8,9,10]. Furthermore, the use of robotic systems helps address the concern of intraoperative radiation exposure to surgeons and operating room staff [11]. In addition to accurate pedicle screw placement, robot-assisted surgery offers several benefits, including enhanced pre-operative planning for scoliosis cases, which facilitates a harmonious construct and easier rod placement, particularly during minimally invasive surgery (MIS) for deformity correction. It also provides trajectory guidance and navigation for TLIF and OLIF cages, along with improved docking for endoscopic surgeries and sacroiliac joint fusion [12]. 

As these technologies become more integrated into surgical practice, it is essential to assess their added value and potential cost savings. In this context, the aim of this study was to provide a comparison between robot-assisted and navigation-based spine surgery, with a focus on surgical outcomes and cost savings, to determine whether robotic systems truly offer superior benefits over navigation-based systems as claimed. Additionally, the study will explore the financial implications of adopting robotic systems in spine surgery, examining the potential cost savings that can be achieved through their use.

## 2. Materials and Methods

**Figure 1:**
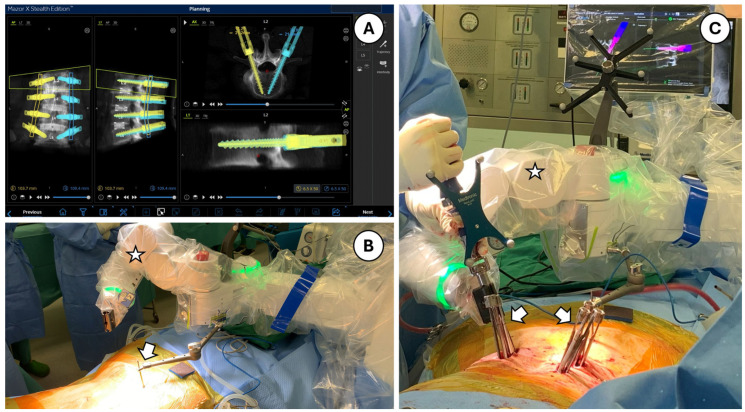
Robot-assisted pedicle screw application: (**A**) surgical planning performed using the Mazor X Stealth Edition robotic software, visualized on the display monitor; (**B**) the robot, connected to the patient via a single percutaneously inserted Schanz screw in the posterior superior iliac spine (arrow); star indicates the robotic arm; (**C**) screw application with real-time monitoring. Arrows indicate minimally invasive screws inserted with robotic guidance; the star represents the robotic arm. The real-time display monitor is visible in the background.

This single-center, retrospective cohort study was conducted at a tertiary hospital following approval by the Domain Specific Review Board (DSRB), National Healthcare Group (NHG), Singapore. Patients who underwent single- and double-level TLIF and OLIF surgeries between October 2022 and May 2024, with pedicle screws placed using robotic assistance, were identified to form the robotic group. Patients who underwent navigation-assisted pedicle screw placements for similar indications by the same surgeon prior to the introduction of the robotic system at the institution, between January 2018 and December 2020, were identified to form the navigation group. Building on the findings from our previous study on the learning curve associated with the use of the robotic system, we excluded the first 20 cases of robotic surgery from the analysis [13,14,15]. This exclusion was made to ensure that the data more accurately reflected the system's performance once the surgical team had gained sufficient experience. The steps of surgery did not deviate from what was previously described (Figure 1). The robot we use (Mazor X Stealth Edition, Medtronic Inc., Dublin, Ireland) offers two distinct operational modalities: CT-Fluoro merge and Scan-and-Plan. We prefer the CT-Fluoro merge method, which allows us to plan the surgery a day in advance using preoperative CT images and merge them with intraoperative fluoroscopy for guiding MIS screw placement using the robot. 

The navigation and robotic groups were stratified based on the type of surgery (TLIF or OLIF) and the number of fusion levels (one or two). The groups were then matched for comparative analysis based on these surgical factors. Data on demographics, comorbidities, diagnoses, operative duration, intraoperative blood loss, and length of hospital stay were collected and analyzed. The mean operative duration, blood loss, and length of stay were calculated for each group. The primary focus of the analysis was to calculate the mean operative duration, the average amount of blood loss during the procedure, and the average length of hospital stay for each patient group under investigation. These metrics were evaluated to determine the efficiency of the surgical process and its impact on patient recovery, with particular attention to the potential for cost-saving measures. Cost savings were quantified by examining two critical factors: a reduction in the operative time and a shorter length of hospital stay. These reductions can translate directly into lower operational and healthcare costs, which are a significant consideration in hospital management.

To calculate the savings from a reduction in operating time, it was necessary to estimate the cost of operating theater use. In our institution, the estimated cost for using the operating theatre is approximately SGD 1500 (Singapore Dollars) per hour. This figure encompasses all associated costs for intra-operative care. Similarly, the daily cost associated with a patient’s stay in the hospital was another important consideration for cost-saving calculations. According to data from the hospital’s value office, the daily cost for a patient’s stay was approximately SGD 1169. This figure encompasses all associated costs for inpatient care. By incorporating these cost estimates, we assessed the total savings for each individual case where robotic assistance was utilized. These savings were extrapolated to estimate the projected cost reductions for the institution depending on the volume of robotic surgeries performed.

All statistical analyses to compare the means of each outcome between the navigation and robotic groups were performed using GraphPad Prism 9 (GraphPad Software Inc., San Diego, CA, USA). To assess the distribution of each group, the Shapiro–Wilk test was conducted to check for normality. For normally distributed data, a two-sample t-test was used to compare the means of continuous variables between the groups. For data that were not normally distributed, the Mann–Whitney U test was applied to compare the groups. To analyze categorical variables, the Fisher's exact test was used. Statistical significance was determined with a threshold of a *p*-value < 0.05, indicating that results were considered significant if the probability of observing the given data by chance was less than 5%.

## 3. Results

This study involved a total of 81 patients, divided into two groups: 45 patients in the navigation group (17 males, 38%; 28 females, 62%) and 36 patients in the robotic group (19 males, 53%; 17 females, 47%). The difference in the sex distribution between the two groups was not statistically significant (*p* = 0.19). The mean ages of the patients in the navigation and robotic groups were 64.8 ± 12 years and 65.8 ± 7.3 years, respectively, with no statistically significant age difference between the two groups (*p* = 0.33). Additionally, the Charlson Comorbidity Index, which assesses comorbidities, showed no significant difference between the two groups (*p* = 0.45) as well. A comprehensive breakdown of the cases within each group, categorized by the type of surgery (one or two levels, specifically TLIF or OLIF) and the surgical approach (open versus minimally invasive), is provided in Table 1. 

The average operative duration, blood loss, and length of hospital stay across the two surgical groups were compared (Table 2). In comparison to navigation-based surgery, robotic surgery demonstrated excellent results, with a statistically significant reduction in operative duration for both one-level and two-level OLIF cases, decreasing by 50 min and 62 min, respectively (*p* < 0.05). To understand whether the observed time savings are due to the use of the robot rather than the position (single vs. dual), we conducted a subgroup analysis, examining single-position and dual-position OLIFs separately. For one-level dual-position OLIF, both the navigated and robotic groups had 10 patients each, while for two-level dual-position OLIF, the navigation group had 13 patients, and the robotic group had 5 patients. This allowed us to compare operative durations, and we found significant time savings in both one-level (*p* = 0.0051) and two-level OLIF cases (*p* = 0.015) using the robot. However, we could not perform such an analysis for single-position surgery due to the smaller number of patients.

These findings underscore the enhanced efficiency and potential time-saving benefits that robotic assistance can provide during spinal surgery. However, despite these improvements, there was no statistically significant difference in operative duration when comparing robotic and navigated two-level TLIF cases. This suggests that while robotic surgery may offer notable advantages, the surgical approach and number of levels operated play a contributing role. 

Other factors that did not reach statistical significance include the reductions in blood loss and length of hospital stay, as observed in Table 2. With robotic assistance, blood loss was reduced by 28.8% for two-level TLIF, 17.0% for one-level OLIF, and 25.5% for two-level OLIF. Similarly, for one-level TLIF, the length of stay decreased by an average of 1.4 days, while one-level OLIF showed a reduction of 1 day, and two-level OLIF had a more substantial reduction of 2.3 days. Although these reductions are notable, their impact is limited due to the lack of statistical significance.

**Figure 2:**
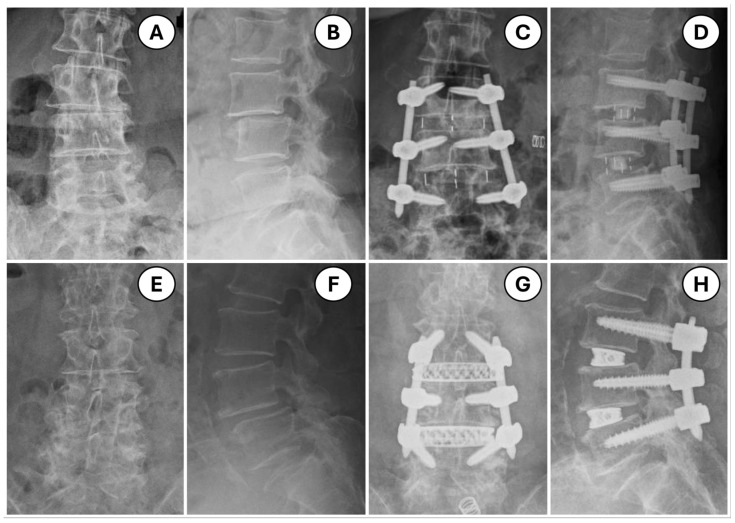
Representative pre- and post-operative radiographs of patients who underwent two-level OLIF with posterior pedicle screw application utilizing navigation or robotic assistance for pedicle screw placement: (**A**–**D**) navigation group; (**E**–**H**) robotic group. (**A**,**C**,**E**,**G**) antero-posterior views; (**B**,**D**,**F**,**H**) lateral views.

Overall, both robotic and navigation-based MIS procedures were successfully performed without the need for conversion to open surgery. This demonstrates the feasibility and effectiveness of both approaches in achieving the desired surgical outcomes. As shown in Figure 2, all screws were placed as intended, and there were no instances of patients needing to return to the operating theatre for revision or correction, further emphasizing the precision and reliability of both robotic and navigation-based systems in spinal surgery. 

At our institution, the estimated cost of utilizing the operating theater is approximately SGD 1500 per hour, factoring in manpower expenses, facility fees, and miscellaneous costs. For two-level OLIF cases, a reduction of one hour in operative duration results in cost savings of SGD 1500 per patient. Additionally, the daily cost to the institution for inpatient general ward stay is SGD 1169; however, we found no significant reduction in length of hospital stay in terms of one or two level TLIF or OLIF cases. Consequently, the total savings per two-level OLIF case using the robotic system amounts to SGD 1500 compared to navigation-based methods. If we perform 200 such cases annually, the total savings will reach SGD 300,000 per year, and SGD 1,500,000 over five years. This cost reduction effectively offsets the initial investment for the robotic system, which is approximately SGD 1 to SGD 1.5 million depending upon the model utilized.

## 4. Discussion

There are numerous advantages to using robotic technology in spine surgery. Robotic systems allow for more accurate planning and execution of screw insertion and interbody cage placement [16]. This is made possible by the robotic arm’s guidance for screw and cage trajectory based on the operative plan, eliminating the factor of human error during insertion. It allows for greater accuracy when operating on cases with spinal deformities [17,18,19] and facilitates the use of MIS techniques, enabling surgeons to perform both lateral or prone single-position OLIF where the cage and pedicle screws can be placed while the patient is in one position [20]. More recently, endoscopic procedures can also benefit from the robotic system’s recent software upgrades [21,22]. Moreover, spine surgery performed with robotic assistance significantly reduces radiation exposure to the surgical team [11]. 

Looking at the literature comparing robotic guided screw placements and conventional methods, a systematic review by Lopez et al. and a meta-analysis performed by Jung et al. both showed robot-assisted spine surgery to have greater pedicle screw accuracy and lower radiation exposure compared to traditional fluoroscopic-guided techniques [23,24]. Jung et al. showed that pedicle screws were 1.1–1.24 times more likely to be placed successfully in robot-assisted surgery compared to fluoroscopic-guided techniques [24]. A comparative study showed a pedicle screw placement accuracy of 93.4% with robotic systems compared to 88.9% with the freehand technique [25], with some studies even showing an accuracy of up to 97.7% with robotic surgery [26]. Robotic surgery allows for greater reproducibility and consistency owing to the precision of robotic guidance, which can be more variable with freehand techniques. One-to-one comparisons of robot-assisted versus navigation-assisted pedicle screw placement are sparse. However, a few studies focusing on accuracy suggest that robotic systems either enhance accuracy or match that of navigation systems. Additionally, newer robotic systems demonstrate significantly lower radiation exposure, reduced robot abandonment rates, and fewer blood transfusions. An important advantage is that these systems do not require real-time trajectory adjustments during the procedure, as is necessary with navigation, which helps mitigate the possibility of human error.

As with any new technology, there are concerns regarding its cost to the healthcare system. While the upfront costs of using newer robotic systems may be higher compared to navigation systems, cost savings should also be considered by evaluating potential reductions in operative duration, blood loss, length of stay, and perioperative complications, as these factors significantly contribute to overall healthcare costs. Few studies have explored the cost-effectiveness of incorporating robotic systems in spine surgery. A study by Menger et al. in the United States healthcare setting demonstrated that robot-assisted spine surgery resulted in fewer revision surgeries, lower infection rates, reduced length of stay, and shorter operative times, ultimately making it a cost-effective option [27]. Building on these findings, our study further emphasized the significant cost savings associated with robotic spine surgery at our center, highlighting its potential to enhance patient outcomes while optimizing resource utilization.

As mentioned earlier, robot-assisted surgeries facilitate MIS approaches [12,16]. This represents a notable advantage, as it enables surgeons to make smaller incisions, resulting in less postoperative pain, reduced blood loss, and faster recovery times for patients. The ability to perform MIS procedures with precision and efficiency would not have been possible to the same extent without the assistance of robotic systems. Our data underscore this paradigm shift in surgical practice, showing that a significant number of one- and two-level OLIF and two-level TLIF cases in the navigation group were performed using an open approach, while all cases in the robotic group utilized an MIS approach. This demonstrates that surgeons can manage more complex cases with MIS using robotic systems, potentially leading to quicker recovery times for patients, which can impact the length of hospital stays and overall costs. The same applies to adopting single-position OLIF surgeries, where patient repositioning for pedicle screw placement can be avoided to save time. Furthermore, screws can be placed simultaneously by another surgeon while the cage is being inserted [20]. Therefore, the integration of robotic systems into spinal surgery offers not only the benefit of increased efficiency but also substantial economic and logistical advantages.

In our study, we observed a statistically and clinically significant decrease in operative duration with robotic surgery for one- and two-level OLIF cases, saving approximately 50 min to an hour on average for each case. This represents a reduction of 16.6% to 17.8% in operative time. Notably, many of our robotic OLIF cases were performed using a single-position approach, while almost all navigation OLIF cases required a dual position during surgery (lateral to prone). Moreover, placing screws from the lateral position using freehand techniques or even with navigation guidance can be cumbersome due to unfamiliarity, potentially leading to higher incidences of breaches and facet joint violations [28]. With the robotic arm, this task becomes easier, allowing screws to be placed with a high level of accuracy while the surgeon is seated and the patient in a lateral position. This capability has encouraged more lateral surgeons to adopt single-position surgery, which may explain the significant decrease in operative duration for these cases.

Robot-assisted screw placement is inherently faster than navigation-based techniques when it comes to the insertion of individual screws. The robotic system’s precision and automation allow for quicker, more accurate screw placement, reducing the time required for each insertion. However, one factor that can extend the overall operative time is the registration process and the setup of the robotic system itself. These initial steps, which involve mounting of the robotic arm, mapping of the patient's anatomy, and calibrating the robotic system to the surgical field, can be time-consuming. As a result, the time savings achieved through robotic screw placement may be somewhat offset by the additional time needed for the system’s preparation, which serves as a limiting factor, particularly in single-level surgeries. The true utility of robots in terms of time-saving becomes more evident in multi-level surgeries. In a multi-level scenario, since the registration process and system setup remain largely the same, regardless of the number of levels being treated or the complexity of the surgical case, as more screws are inserted, the time saved with each insertion accumulates, ultimately leading to a significant reduction in overall operative time compared to traditional methods.

When computing the cost savings of robotic surgery, it is important to consider not only the expenses associated with using robotic technology but also the time spent in the operating theatre and the length of hospital stay. Time in the operating theatre directly impacts healthcare costs, as longer procedures generally lead to higher expenses for the institution due to extended use of resources, including surgical staff, equipment, and the opportunity cost of not being able to perform other surgeries. Additionally, the length of hospital stay influences overall costs due to daily ward charges and potential complications arising from prolonged hospitalization. These factors should be evaluated when assessing the cost savings of robotic surgery, as reducing operative time and improving recovery periods can ultimately lead to more cost-effective care and better patient outcomes. 

As mentioned earlier, the use of robotic systems in two-level OLIF procedures offers cost savings by reducing operative time, with an estimated saving of SGD 1500 per patient. Over five years, performing 200 such cases annually could yield a total savings of up to SGD 1.5 million, which could offset the initial investment for buying the robot. It is important to note that we intentionally kept this estimate on the lower end, as institutions with higher caseloads and more spine surgeons could achieve even greater savings through shared utilization of the robotic system. Furthermore, the cost savings for three or more level cases are likely to be even greater due to a more substantial reduction in operative duration, resulting in higher overall savings. In such cases, we also estimate a significant reduction in the length of hospital stay, factoring in the MIS nature of the surgery. Therefore, robotic surgery can lead to cost-saving than navigation-based methods when considering the resources conserved through reduced operative times and length of hospital stay.

The strength of this study lies in its design as a single-surgeon series, which ensured consistency in the surgical techniques and intricacies employed across patients. This consistency allows for reliable comparisons with the available data. However, as a retrospective study, it has certain shortcomings. Firstly, the comparative analysis was not matched for surgical approach (open vs. MIS), as there were no patients in the robotic group who underwent surgery using an open technique. Therefore, the favorable outcomes associated with robotic surgery may be attributed to the greater use of MIS techniques. However, it is important to note that robotic surgery facilitates this increased adoption of MIS approaches, which may not be practical without the robot. Thus, this can be considered a benefit of robotic surgery, supported by this study. Another limitation of our study is its exclusive focus on surgeries involving up to two levels. We believe that robotic surgery demonstrates greater utility as the number of levels increases, since only a single registration is required regardless of the number of screws placed. This efficiency results in time savings with each additional screw, potentially leading to reduced lengths of stay that may not be reflected in this study. Therefore, further research is recommended to evaluate the outcomes of surgeries involving three or more levels. Lastly, our study did not assess the accuracy of screw placement, as patients do not routinely undergo postoperative CT imaging. However, there were no instances of patients requiring a return to the operating theatre due to implant malposition.

## 5. Conclusions

Robot-assisted spine surgery presents several advantages over traditional navigation-based surgery. In this retrospective study comparing robot-assisted and navigation-based surgeries in patients undergoing one- and two-level TLIF and OLIF, we observed significant reductions in operative duration for robot-assisted one- and two-level OLIF surgeries, with average time savings of 50 and 62 min, respectively. These time savings correspond to an estimated cost reduction of SGD 1500 per patient for a two-level OLIF procedure, with the potential for even greater savings as the number of levels increases. The findings suggested that robot-assisted spine surgery offers superior efficiency and cost savings, particularly in cases with more surgical levels. As robotic technology continues to advance, its integration into spine surgery becomes increasingly justified, offering improved patient outcomes and enhanced cost-effectiveness.

## Figures and Tables

**Table 1:** Breakdown of navigation and robotic cases by type of surgery and surgical approach.

Procedure	Approach	Navigation (*n* = 45)	Robotic (*n* = 36)	Total (*n* = 81)
1 level TLIF ^a^	Open	0	0	0
	MIS ^b^	12	9	21
2 level TLIF	Open	4	0	4
	MIS	5	4	9
1 level OLIF ^c^	Open	4	0	4
	MIS	7	15	22
2 level OLIF	Open	5	0	5
	MIS	8	8	16

^a^ Transforaminal lumbar interbody fusion; ^b^ minimally invasive surgery: ^c^ oblique lumbar interbody fusion.

**Table 2:** Comparison of surgical efficiency between navigation and robotic surgical groups.

Outcome of Interest	Procedure	Navigation Group	Robotic Group	Difference Between Means	*p*-Value
Operative Duration (min)	1 level TLIF ^a^	208 ± 33 min	230 ± 45 min	−22 min (−10.6%)	0.23
	2 level TLIF	322 ± 41 min	317 ± 49 min	5 min (1.8%)	0.85
	1 level OLIF ^b^	280 ± 39 min	230 ± 39 min	50 min (17.8%)	0.0004
	2 level OLIF	376 ± 62 min	314 ± 59 min	62 min (16.6%)	0.0354
Blood Loss (mL)	1 level TLIF	104 ± 49.8 mL	171 ± 160 mL	−60 mL (−57.6%)	0.60
	2 level TLIF	233 ± 122 mL	167 ± 50 mL	67 mL (28.8%)	0.45
	1 level OLIF	141 ± 74 mL	117 ± 126 mL	24 mL (17%)	0.29
	2 level OLIF	185 ± 90 mL	138 ± 106 mL	47 mL (25.5%)	0.25
Length of Stay (days)	1 level TLIF	7.5 ± 5.9 days	6.1 ± 4.2 days	1.4 days (18.7%)	0.64
	2 level TLIF	10.2 ± 5.6 days	9.3 ± 4.6 days	1 day (9.8%)	0.75
	1 level OLIF	6.6 ± 3.4 days	6.9 ± 5.2 days	−0.2 days (−3%)	0.46
	2 level OLIF	9.4 ± 4.3 days	7.1 ± 2 days	2.3 days (24.5%)	0.30

^a^ Transforaminal lumbar interbody fusion; ^b^ oblique lumbar interbody fusion; a *p* value of < 0.05 is considered statistically significant.

## Data Availability

The data presented in this study are available upon request from the corresponding author due to ongoing studies utilizing the dataset.
